# Role of EMT in Metastasis and Therapy Resistance

**DOI:** 10.3390/jcm5020017

**Published:** 2016-01-27

**Authors:** Bethany N. Smith, Neil A. Bhowmick

**Affiliations:** Department of Medicine, Cedars-Sinai Medical Center, 8750 Beverly Blvd., Atrium 103, Los Angeles, CA 90048, USA; bethany.smith@cshs.org

**Keywords:** EMT, microenvironment, stroma, prostate cancer

## Abstract

Epithelial–mesenchymal transition (EMT) is a complex molecular program that regulates changes in cell morphology and function during embryogenesis and tissue development. EMT also contributes to tumor progression and metastasis. Cells undergoing EMT expand out of and degrade the surrounding microenvironment to subsequently migrate from the primary site. The mesenchymal phenotype observed in fibroblasts is specifically important based on the expression of smooth muscle actin (α-SMA), fibroblast growth factor (FGF), fibroblast-specific protein-1 (FSP1), and collagen to enhance EMT. Although EMT is not completely dependent on EMT regulators such as Snail, Twist, and Zeb-1/-2, analysis of upstream signaling (*i.e.*, TGF-β, EGF, Wnt) is necessary to understand tumor EMT more comprehensively. Tumor epithelial–fibroblast interactions that regulate tumor progression have been identified during prostate cancer. The cellular crosstalk is significant because these events influence therapy response and patient outcome. This review addresses how canonical EMT signals originating from prostate cancer fibroblasts contribute to tumor metastasis and recurrence after therapy.

## 1. EMT Classifications

**Figure 1:**
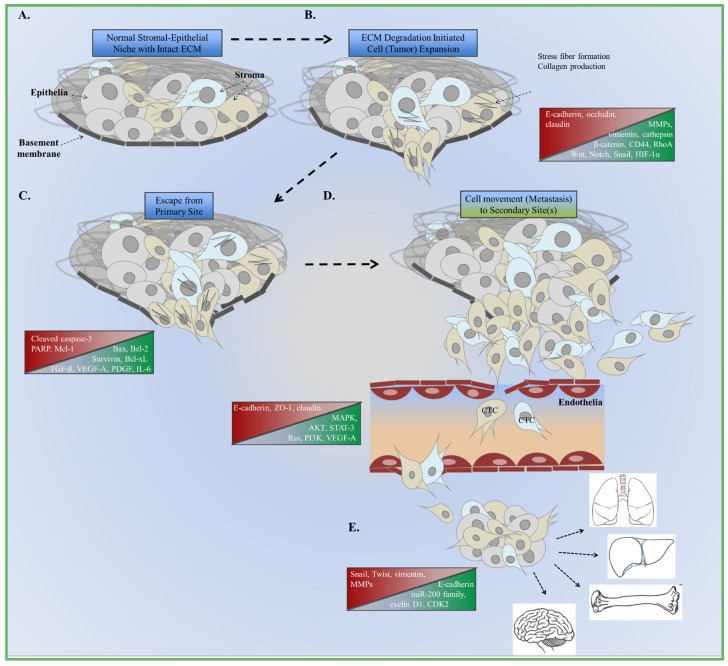
Summary of EMT and metastasus. (**A**) Normal interactions between cells and extracellular matrix (ECM) compobnents. Changes in canonical signaling and cellular function are the foundation of aberrant cell growth and expansion; (**B**) Increased expression of enzymes that degrade the ECM are involved in decreasing adhesion to the basement membrane; (**C**) Cells escape from the basement membrane as growth factor and cytokine signaling increases to accommodate changes in the microenvironment; (**D**) Cells intravasate and spread to the bloodstream as circulating tumor cells (CTC); (**E**) Extravasation at the secondary site promotes the formation of micrometastases and the re-induction of epithelial markers (MET). Sites of metastasis include the lungs, liver, bones, and brain.

The steps of EMT include a combination of the microenvironment molecules adjusting to accommodate cellular expansion to either promote tissue development in physiological or pathological contexts [1,2,3,4,5]. These changes depend on spatial and temporal cues, characterized by the production of secretory enzymes to degrade the extracellular matrix (ECM), while cell expansion continues [5]. ECM degradation by matrix metalloproteinases (MMPs) is a clear example of this step of EMT. Furthermore, transforming growth factor-beta (TGF-β) regulates MMPs and downstream Smad signaling to enhance these degradation activities. Steps of EMT in cancer are comparable when cells intravasate into the circulatory and/or lymphatic systems, extravasate at the secondary site, and micrometastases develop during advanced disease [6,7,8]. These phenotypic changes illustrate the cancer cell plasticity required for tumor escape. To clearly discuss the molecules that control EMT and potential therapeutic approaches, EMT classifications as type-1, type-2, and type-3 are explained [9,10,11]. These types of EMT seem to be based on a combination of several factors, including components of the microenvironment, the context of the epithelial and stromal cells, and exogenous changes in host immunity. More specifically, stromal fibroblast “re-education” can be used to study adjacent epithelial cells. The transformation of the stromal compartment along with microenvironment changes provides a niche, where morphological signals influence tissue vitality and integrity (Figure 1A). Type-1 EMT is associated with embryogenesis and normal tissue development, type-2 with wound healing, and type-3 with tumor development and metastasis [12]. Type-1 EMT meets a terminal point during fetal development and after birth in contrast with type-2 and type-3 EMT. Embryo development requires the transformation of neural crest cells to generate three defined germ layers, responsible for physiological growth and expansion. Epithelial, mesenchymal, and mesodermal cells function during this process. Mesenchymal cells only exhibited migratory and invasive properties based on high expression of vimentin, *N*-cadherin, and low expression of *E*-cadherin. Wnt signaling orchestrates these steps of EMT based on findings where Wnt-deficient embryos lost their ability to undergo gastrulation. Similar results were observed when ectopic expression of Wnt8c in embryos induced primitive streak formation [13]. The epithelia also serve as the foundation for renal fibrosis where morphological changes define the clinical outcome [14]. The role of TGF-β during these steps control Wnt along with other regulators, such as Nodal and Vg1 [2]. Transcription factors related to type-1 EMT include mesodermal posterior 1 and 2 (Mesp1, Mesp2) during gastrulation [15], Snail [16,17], and eomesodermin (EOMES) [18] while downstream target gene transcription is either repressed or activated. Snail overexpression in ARCaP_M_ cells influenced the ECM by decreasing the expression of integrins α5, α2, and β1 [16]. These effects were also similar when Snail transcriptional repressed maspin in 22Rv1 prostate cancer cells [17]. Snail knockdown reversed these effects to reduce EMT in prostate cancer cells. The common mesenchymal phenotypes at the initiation of embryogenesis generate diverse cell types based on these molecular interactions. Wnt signaling can regulate Snail activity to promote embryonic stem cell renewal or differentiation [19,20,21]. Snail conditional knockout experiments in embryonic stem cells had activated Wnt3a and Axin2 expression compared to control cells [19]. Wnt inhibition did decrease Snail activity, but Snail knockout did not affect EOMES expression. Traditional patterns of high Snail expression correlated with low *E*-cadherin expression were reversed after Snail knockout studies. These findings clearly demonstrate this molecular link during normal development as these cells also possessed cuboidal (epithelial) morphology. Furthermore, proliferation and cell death signals were unaffected after Snail knockout. A link to type-2 EMT can be seen when Snail did not affect embryonic stem cells migration after *wound healing* assays were performed [19]. Mesp1 induces mesoderm differentiation via Snail in embryonic stem cells, and have been studied in regards to miRNA regulation. miR-200 is downregulated when Snail is activated to enhance pluripotency (miR-294, miR-295, miR-292-3p) and repress the epithelial phenotype (miR-200c, miR-200b, miR-429), another feature of enhanced EMT [22,23]. TGF-β signaling inhibitor SB-431542 stopped activin receptor-like kinase 5 (activin) and miR-200 functions to promote EMT and differentiation. These features are important to note based on the function of activin during wound healing and skin carcinogenesis [12]. However, there is a balance of activin required to repair damaged tissues and prevent scar tissue formation that may lead to skin tumors.

**Figure 2:**
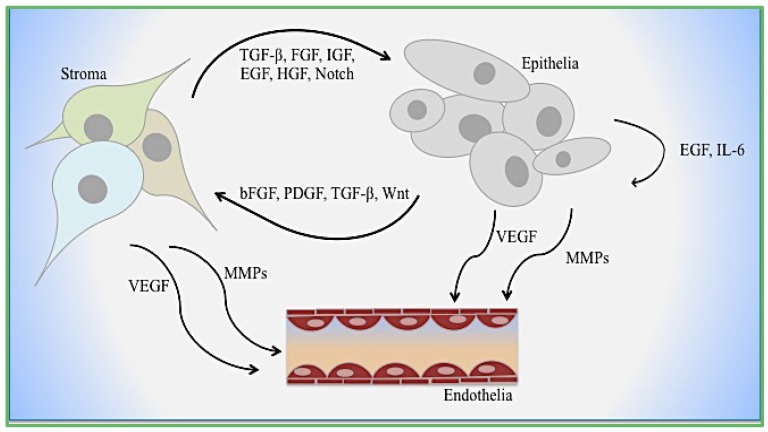
Cellular interactions during EMT. Mechanisms of epithelial–stromal–endothelial crosstalk are depicted. Growth factors, cytokines, and enzymes function dynamically with and between each cell type.

Cancer tissue remodeling associated with wound healing, described more than 150 years ago by Virchow, is a major component of the EMT mechanism [10]. Similarities between type-2 and type-3 EMT are found within the stroma of wound healing and transformed tumor cells. Cellular shape and extracellular matrix deposits from the stroma include collagen, which contributes to microenvironment remodeling (Figure 1B). Tenascin-C, fibronectin, and fibrinogen increase to accommodate these steps as cell shape changes [24,25]. The same markers used to monitor the changes of cell morphology and function is recognized within the circulatory system. Subsequently, the secretion of these proteins serves as the substrates to promote survival and migration [26,27] (Figure 1 and Figure 2). Claudins, occludins, and E-cadherin serve as these substrates during EMT to deregulate the ECM and microenvironment.

Development of cardiac tissue requires type-1 EMT in the generation of cardiac valves from endocardial cushions, whereas type-2 EMT is involved in adult cardiac disease and other diseases of the kidney and liver [14,28,29,30]. Disruption of normal myocardial tissues is regulated by adjacent fibroblasts. These fibroblasts recruit extracellular matrix (ECM) molecules and originate from surrounding endothelial (blood vessel) cells. These activities demonstrate a connection between type-1 EMT and endothelial EMT (endo-MT), another mechanism promoting support for expanding tissues [31,32]. TGF-β induced the development of fibroblast cells during type-1 and endo-MT. Conversely, bone morphogenetic protein 7 (BMP-7) maintained the endothelial phenotype, inhibited endo-MT and progression of cardiac fibrosis, renal injury, and colon cancer [33,34,35]. The expanding fibroblast cells involved in cardiac development invade and assist in epicardium formation, and maintain structural integrity observed in the adult mesodermal tissues [36,37]. Similar observations have been made in renal pathologies [9,33,38,39], liver fibrosis [30], and during gastrulation and neural crest formation [36].

Type-2 EMT is activated during injury, and has been studied during kidney fibrosis [40]. The mechanism of fibrosis can be monitored or predicted using specific fibroblast markers for cell morphology. Kidney fibrosis cells expressed fibroblast-specific protein 1 (FSP1) and conducted EMT, demonstrated by fibroblast phenotypes [41]. FSP1 is a filament-associated, calcium-binding protein specifically expressed at high levels in cells with the EMT phenotype. Findings also indicated that sometimes fibroblasts arise from the local conversion of epithelial cells [29]. NP-1 tubular epithelial cells submerged in type I collagen gels converged to a fibroblast phenotype accompanied with *de novo* FSP1 and vimentin expression. After developing a polyclonal FSP1 antibody, expression was also noted in kidney, lung, and spleen tissues. Embryonic analysis showed that *FSP1* was not expressed in the embryo at day E8.5, although mRNA could be detected in decidual tissues from the uterus [3]. A monoclonal antibody to the β-subunit of the PDGF-receptor was used to determine expression in renal tissues [42]. Mesangial and peritubular interstitial cells within the glomerulus have high PDGF-receptor expression during glomerulonephritis, and cellular shape is similar to EMT. FSP1 is selective for cells exhibiting the fibroblast phenotype, unlike previous markers used to study injury and healing [43]. Platelet-derived growth factor (PDGF) [42], α-smooth muscle actin (α-SMA) [43], and ecto-5′-nucleotidase [44] expression was associated with myofibroblast accumulation during injury. The elevated expression of FSP-1 in kidney fibrosis helped support tubular epithelia cells EMT transformation in generating interstitial fibroblasts during kidney injury [28] and fibrosis [29]. FSP-1, for example, is regulated by a fibroblast transcription site (FTS-1) that exists in the promoters of EMT-promoting genes such as Twist, Snail, beta-catenin [42]. These activities correspond to fibroblast-regulated fibrosis in intestines and pulmonary tissues [45]. It is important to note other sources of myofibroblasts in tumor tissues include pericytes, resident fibroblasts, as well as bone marrow-derived mesenchymal stem cells [46,47]. Fibroblasts associated with lung cancer have a clear expression profile related to their involvement during tumor progression. The microarray gene expression of proteins regulated by TGF-β and MAPK signaling was higher in cancer-associate fibroblasts (CAF) cells compared to normal fibroblasts [46]. Similar to findings from Neal *et al*. [16], integrin α11 was higher in CAF cells following microarray analyses.

TGF-β signaling was analyzed in NMuMG mouse mammary gland cells where TGF-β treatment induced epithelial differentiation [48]. Although these cells express elevated TGF-β type I receptor Tsk7L, they lack expression of ALK-5/R4 type I receptor. A shortened form of the Tsk7L type I receptor inhibited EMT in the presence of TGF-β, even with responsiveness. Cell growth and E-cadherin were reduced indicating a role for type I TGF-β receptors. Furthermore, these activities occur in a RhoA-dependent manner in NMuMG cells [49]. Type-3 EMT is identified during cancer development and metastasis—the primary focus of this review. TGF-β is a master regulator of cancer development, where decreased *E*-cadherin, ZO-1, and desmoplakin 1 expression in breast epithelial cells correlated with a fibroblast-like morphology [50]. Actin fiber reorganization occurred after TGF-β treatment, representing an early sign of EMT. This event was characterized by cellular differentiation [51], also indicated in another study where breast tumor EMT was regulated by TGF-β and RhoA [49]. These breast epithelial cells had the classical EMT morphology and expression of mesenchymal markers. This study also indicated that TGF-β-induced EMT was unchanged even when Smad signaling decreased, and stress fiber formation regulated by TGF-β was not Smad-dependent. TGF-β, p38MAPK [50], and AKT signaling interactions were found to be significant for tumor-specific EMT [52,53], as well as TGF-β ligand [54,55], and BMP-7 [34,39]. TGF-β signaling in the fibroblasts regulated adjacent tumor epithelia to promote prostate cancer [56]. These observations indicated a role of this cell type to regulate oncogenic potential in epithelial cells using a fibroblast-specific TGF-β receptor 2 knockout model (Tgfbr2fspKO). Alternatively, TGF-β ligand BMP-2 induces invasion and cancer stem features via STAT3 activation during colon cancer [35], and ERK1/2 and AKT during gastric cancer [57,58,59]. Tissue scarring depicted by fibrosis, followed by the administration of tumor necrosis factor alpha (TNF-α), leads to mesenchymal marker expression [60]. Migrating epithelial cells of human skin had increased vimentin and FSP1 expression. Cells contained within hypertrophic scars also had increased mesenchymal marker expression, combined with inflammatory cytokine expression. Expression of MMPs, FSP1 and vimentin were induced after TNF-α treatment antagonized by targeting BMP2/4. These findings demonstrated similarities during wound healing to promote EMT. Breast cancer EMT has been studied using fibroblast growth factor-10 (FGF-10) [61]. FGF-10 treatment increased tumorigenesis by increasing the MDA-MB-231 cell ability to migrate as indicated by wound healing assays, decreased apoptosis, and increased Wnt signaling. Cells lining the blood vessels supplying the tumors with nutrients, and a preferred environment to develop micrometastases characterize endo-MT. Endo-MT is observed to have a significant role in all three types of EMT. The endothelia serve as precursors for hematopoietic cells during embryonic development (type-1), fibrosis (type-2), and cell malignancy (type-3) in the adult [3,15,26]. Most of the cell types within the circulatory system develop according to the Endo-MT cues responding to factors like platelet-derived growth factor (PDGF), periostin, and several within the Notch signaling pathway. Studies focused on the signaling and cross-talk between different cell types during tumor progression have relied on the functionality and preference of tumor cells to respond to the fibroblast-specific cues, especially during later stages of tumorigenesis. Proliferative endothelia have been associated with the proliferations and motility of adjacent breast cancer epithelia [27,62]. Cancer cells associated with EMT promote expansion of mesenchymal cells where poor patient outcome was correlated with elevated Twist, Snail and CD44 expression in lung cancer [63]. Since tumor vasculature can be critical to the expansion of tumors, type-1 EMT in the form of endo-MT can play a paracrine role in tumorigenesis. Together, these can be a mechanism for the expansion of CAF.

## 2. EMT Mediators: Tumor and Microenvironment Crosstalk

Conserved zinc finger transcription factors (Snail, Zeb-1, Slug), regulated by upstream TGF-β activities, have been shown to drive EMT functions [64,65,66]. Snail also represses *E*-cadherin transcription at the promoter level by binding to its enhancer box sequence. These functions are related to the differentiated morphology to subsequently promote tumor metastasis. High expression of these types of molecules compared to non-tumor molecules are correlated with poorer prognosis in clinical analyses, as observed with Snail and Twist in lung adenocarcinoma [63], Slug in gastric cancer [64], Snail in prostate cancer [16,17] and breast cancer [67,68]. More specifically, the epigenetic events as described with zinc finger protein methylation of ZBTB20 [66], ZNF139 [69], and ZNF545 [70] are associated with tumor metastasis. Recent findings indicate that pancreatic tumor cells are able to conduct EMT in the absence of Snail and Twist transcription factors [71]. These cells were able to conduct metastasis even after Snail or Twist knockout. *In vivo* analysis of Snail- or Twist-induced EMT was not rate-limiting for invasion and metastasis in transgenic mouse models. Yet, *in vitro* overexpression of Snail regulates maspin tumor suppressor transcription in prostate cancer [17], and estrogen receptor alpha [67] and kinase activities in breast cancer cells [53]. The regulatory activities of Snail and Slug were also demonstrated when they activated TGF-β in breast cancer cells [68]. The data indicate a contrast between the physiologically relevant and cell culture conditions that are significant to EMT during cancer. Commonalities between the *in vivo* and *in vitro* conditions contributing to EMT are regulated by TGF-β, MAPK, and ECM component activities [72]. The fibroblast phenotype of these EMT tumor cells is critically important based on analyses of the surrounding microenvironment. CAF and tumor epithelial cells coordinate during EMT to promote vascular intravasation [73] (Figure 1). Circulating tumor cell (CTC) can escape from primary radiation or surgical intervention and their capture by current methods rarely indicate their potential to engraft and expand in a secondary metastatic site.

EMT is enhanced in part due to the maintenance of the fibroblast morphology and intricate crosstalk with the tumor microenvironment, also related to decreased cell adhesion [74]. Molecules that compose tight junctions, ECM components [75], and fibroblast-derived secretome [76] are examples of the crosstalk between the tumor and microenvironment. RhoA signaling is a traditional mediator of morphologic change. RhoA-ROCK signaling can serve as a switch to enable cell cycle arrest [49]. A general theme that has emerged is that proliferation and differentiation do not occur at the same time. There is controversy in the role of Smad proteins in the progression of EMT downstream of TGF-β. Smad2/3 may not be essential for morphologic changes of the epithelia, but it is a required component of cell cycle arrest for transdifferentiation steps to progress. These changes include cytoskeletal changes depicted by shifts in polarity due to actin reorganization in metastatic cancer cells [77], and increased motility [75,78]. Cytochalasin D (Cyt D) treatment of breast cancer cells increased E-cadherin and reduced RhoA expression. Remodeling of the cytoskeleton plays a crucial role in EMT to promote cancer. *In vivo* analysis indicated that vimentin was 3.7 times higher compared to *E*-cadherin in oral squamous cell carcinoma [78]. Wnt, Notch, and TGF-β signaling can additionally potentiate the expression of Zeb1, FoxC2, and Twist for mesenchymal morphologic changes mediated by the inhibition of *E*-cadherin expression and increased expression of vimentin and N-cadherin.

Transformation of mammary epithelial cells has been attributed to the functions of stroma, as compared to findings in prostate cancer [79]. The loss of stromal TGF-β responsiveness enables paracrine hepatocyte growth factor (HGF) signaling in adjacent epithelia tumors, characterized by the fibroblast phenotype [80,81]. Epithelial expression of the c-Met and Ron, HGF cognate receptors, support Stat5, JAK, and TAK1 to promote Snail expression. Platelet-derived growth factor (PDGF) is also identified as a functional determinant for CAF [4,82]. Following an orthotopic mouse model of colorectal cancer, the stroma cells required stanniocalcin-1 (STC1), a PDGF regulator, to promote intravasation of adjacent tumor cells [82]. Thus, epithelial tumors contained within invasive fronts may be dependent on paracrine signaling. Further, when associated with type 3 EMT, the morphologic changes can be associated with cell transformation [83,84]. There is clearly an intricate balance of the intracellular and extracellular activities that modulate EMT and in turn cancer initiation and progression. The cadherin and integrin switches during EMT involve relocation of the cells from the basement membrane environment to the fibrillary ECM zone [85]. Matrix metalloproteinases (MMPs) and integrins function with the tumor microenvironment to induce changes during EMT [62,86,87]. FSP1, matrilysin and stromelysin-1 are EMT-promoting genes that can downregulate E-cadherin to promote EMT [87,88]. MMP-3 and MMP-9 originating from the tumor microenvironment, tumor cells, and stromal cells adjacent to the tumor cells help to facilitate EMT via invasion and metastasis behaviors. The activation and expression of integrins also facilitate interaction with the tumor environment. Overexpression of α_v_β_6_ integrin activated by MMP-3 in poorly differentiated squamous cell carcinomas gained a fibroblastic morphology to EMT during oral cancer [89] and melanoma [90]. Myosin filaments, restructured to accommodate changing cell shape by vimentin and beta-catenin signaling contribute to the invasive front of the tumor. These functions are specifically significant in head and neck squamous cell cancer (HNSCC), and oral squamous cell cancer (OSCC) [91]. Expression of a CD44^high^/CD24^l0w^ protein signature, aldehyde dehydrogenase (ALDH1), Nanog, and stromal cell-derived factor-1/CXCR4 signaling regulate bone metastasis [92]. These activities regulate *cellular plasticity* to enhance the interactions between epithelial and fibroblast cell types.

## 3. EMT and Tumor Metastasis

Although the function of EMT in development has not been disputed, its role in cancer metastasis has long been a point of controversy. EMT is a resultant of paracrine signaling by stromal-derived factors as well as a potential source of cells in the stromal compartment (Figure 2). The difficulty in proving that adult patient tissues unequivocally have cells undergoing EMT is the source of controversy. However, the process of EMT, either as a result of paracrine induction or inherent somatic mutations, can be associated with the gain of stem/progenitor cell differentiation. The gain of the fibroblastic phenotype is often associated with the gain of stem features, such as markers previously described, reduced proliferation, adaptability to secondary microenvironments during metastasis, and therapeutic resistance. The aberrant activation of Ras/mitogen-activated protein kinase (Ras/MAPK) is a feature of cancer stem cells in the pancreas and breast has long been recognized yet it is a difficult target to drug. In prostate cancer, epithelial expression of receptor activator of NF-κB ligand (RANKL) in an osteomimicry phenotype conveys the advantages of osteoclast activation in the bone microenvironment [93,94]. Further profile analysis of bone-specific proteins osteopontin (OPN), osteocalcin (OC), bone sialoprotein (BSP), and osteoprotegrin (OPG) demonstrated expression in prostate cancer epithelia [95,96,97]. Further, the ability of EMT cells to engraft in the bone marrow niche that also supports hematopoietic stem cells is reported. Survival within the skeleton during prostate cancer progression is supported by several growth factors related to TGF-β, ECM, and hormone-regulated receptors. The molecular crosstalk between the prostate tumor and bone stroma are targets of therapies to increase tumor cell death.

EMT was associated with RANKL activation in ARCaP_M_ and LNCaP prostate cancer cells accompanied with Snail overexpression [94]. Similarly, Snail expression in the nucleus of epithelial ovarian tumors increased from precursor lesions to carcinomas that are especially resistant to traditional chemotherapy [98]. In this respect, the stem/progenitor state can mean the cancer epithelial may lose contact inhibition as well as become resistant to anoikis. This line of reasoning can help attribute EMT to these processes of metastasis while identifying transient cells in human primary tissues. The evidence of cells in CTC studies that simultaneously have epithelial and fibroblastic markers can support the role of EMT in the process of metastasis.

The controversial role of EMT has been noted during the regulation of the cytoskeleton protein complexes [99]. Here, it introduced the ability of cells to metastasize in the absence of an overt need to undergo EMT, through a process where a group of cells enter the leaky tumor vasculature in a raft-structure. Integrin outside–in signaling is evident in ECM remodeling by fibroblasts to provide an environment for invading tumor epithelia [100]. Cytoskeleton regulation of Rho GTPases Cdc42, Rac1, and RhoA were challenged using inhibitors. The findings from this study implicated that there are different modes of motility regulated by RhoA/ROCK-independent EMT mechanisms. Tumor cells were able to overcome inhibitors that target motility by switching to a different mode (Rac1 to RhoA and RhoC). Tracks of movement were detected as fibroblasts led epithelial tumors through the ECM. Subsequently, it is presumed that the group of cells could lodge in narrow blood vessels and expand without the need to extravasate. Histologic documentation of similar structures in the bone marrow and lung vasculature support this clinical metastatic possibility. In light of the plasticity of cancer epithelia, both EMT and collective migration methods are both likely modes of metastasis.

## 4. Cooperative EMT Signaling Mechanisms

A common signaling component of multiple paracrine factors including FGF, HGF, and PDGF signaling pathways is Ras activation. Even in the absence of somatic Ras mutations, paracrine Ras activation in the context of TGF-β is a long-recognized cooperation during tumor EMT. Oft *et al.* reported that EpH4 mammary epithelial cells transformed by mutant Ras signaling underwent EMT [101]. These cells possessed a fibroblast phenotype that directly corresponded to their metastatic behavior and resistance to TGF-β1 cell cycle inhibition. The cooperation of TGF-β and Ras signaling can be through multiple signaling proteins, including phosphatidylinositol-3 kinase (PI3K) and mitogen-activated protein kinase (MAPK), to result in the activation of Snail-downstream genes. Antagonizing the MAPK pathways with U0126 in ARCaP cells was able to partially reverse EMT through the downregulation of Snail [16]. TGF-β cooperates in this process by activating integrin signaling significant to epithelium, but not specific to lung tissue [102]. Forsyth *et al.* described EMT as a process that leads to the formation of stromal cells that progress in tandem with invasive carcinoma cells [103].

The various CTC capture technologies suggest, however, that cancer cells that have undergone EMT are in fact in circulation (Figure 1D). This observation can be explained by the gain of somatic mutations, bypassing the need for integrin signaling or additional mechanisms of EMT is the other obvious alternative. Integrin proteins are major players in the TGF-β-dependent tumor metastasis process [102,104,105]. Desmosomes and integrins relate consistently to TGF-β, Wnt, and Notch signaling by regulating not only cellular morphology and composition, but also increasing the potential for intravasation and extravasation during metastasis [106]. TGF-β potentiates integrin expression. However, integrin expression can in turn cooperate with TGF-β signaling. Studies in breast epithelia demonstrated that EMT progression by TGF-β can be interrupted by antagonizing integrin β1. Both integrin β1 and TGF-β have a common downstream signaling target, p38MAPK [50]. However, TGF-β activation of p38MAPK was lost if integrin β1 was neutralized. Since p38MAPK is required for TGF-β-mediated EMT to progress, these findings have interesting implications for epithelia that are non-adherent and possibly found in circulation. Presumably, the reduced outside–in integrin signaling by non-adherent cells could result in mesenchymal to epithelial transdifferentiation [49,105]. There are clearly multiple mechanisms of EMT initiation; however, the means of maintaining the mesenchymal state is not clear. Such understanding can support therapeutic limitations faced in treating cancer cells that undergo EMT.

## 5. Role of EMT in Therapeutic Resistance

EMT is associated with therapy resistance and tumor recurrence. Morphological changes observed during EMT are defined at distinct stages of differentiation. Invasive behavior carried out by tumor epithelia are regulated by the tumor-associated stroma [107]. The persistent accumulation of these cells during the recurrent phase following therapy presents an obstacle during clinical management of cancer. Research findings have long noted that tumor fibroblasts also contribute to therapy resistance. The role of EMT in this context during the reduction of therapy efficacy is evident in several tumors, including prostate and pancreas [108,109,110]. Neuroendocrine differentiation in prostate cancer cells was regulated during hormone treatments, but inadvertently resulted in resistance. These findings are particularly interesting, considering the role of Snail EMT marker during NED in LNCaP prostate cancer cells [111]. MAPK signaling and IL-6-induced neuroendocrine differentiation was increased in LNCaP prostate cancer cells, similar to activities observed in patients undergoing ADT [112,113,114]. It is important to note the fibroblastic and spindle-shaped cells proliferating during these studies. Canonical signaling regulated by MAPK and PI3K/Akt have also regulated EMT to promote resistance and tumor recurrence.

The stem features acquired in the specific EMT process can be of particular importance when considering therapeutic intervention. The activation of Notch signaling was associated with increased cell proliferation and survival in gemcitabine therapy resistance [111]. Similarly, Slug and Snail expression is associated with radio-resistance and chemo-resistance by reducing apoptosis while increasing stemness [99]. STAT-3 signaling (closely associated with IL-6) was antagonized using siRNA in metastatic PC3 prostate cancer cells [114]. The expression of anti-apoptotic gene Bcl-2, cyclin D1 and c-Myc were reduced, indicating a correlation between this signaling pathway and prostate cancer progression. Standard chemotherapy methods used to target cancer, can promote EMT where methods of reversing EMT had presumed benefit [115,116]. More specifically, resistance to therapies such as paclitaxel was associated with increased migration and invasion of tumor cells. Twist not only induces EMT, but increases therapy resistance in breast cancer cells via downregulation of estrogen receptor-α [117] and increased Akt [115]. Targeting the proteasome has also been shown to reduce chemoresistance via Snail signaling. Proteasome inhibitor NPI-0052 downregulated Snail and NF-kappaB expression while Raf-1 kinase inhibitory protein (RKIP) expression increased [116].

Tumor complexity is perpetuated by the communication between tumor cells and the microenvironment, contributing to therapy resistance [118]. Therapies targeting endothelial, cancer stem, and immune-evasive cells also present challenges when tumor signaling adapts. Doxorubicin therapy induced thymic endothelial cell expression of IL-6 and tissue inhibitor of metalloproteinase 1 (TIMP-1), providing tumor cells with a protective niche within the thymus tissue [118]. Following the administration of therapies, hypoxic tumor areas increase tumor growth and survival. The reason that a broader therapeutic strategy of targeting both the tumor epithelia and its microenvironment is starting to show promise may be because cells undergoing EMT are addressed as well as the source of paracrine mediators of tumor progression/differentiation. Tumor metastasis brings about another layer of complexity as the tumor microenvironment changes relative to the primary site. Tumor adaptation to the surrounding environment is influenced by selective pressure applied by drug therapy [119]. Claudin5 (CLDN5) is a potential target for antiangiogenic therapy because it was highly expressed in vascular endothelial cells [120]. Several other CLDN genes (1, 3, 4, 7, 10, 16) were highly expressed in several human tumors. Unfortunately, the long-term negative consequences tend to overshadow the short-lived efficiency of CLDN5 antagonism, as a result of upregulation of other tumor-promoting pathways [121]. Wide-spectrum therapies that are designed to impact more than one pathway or process have had greater efficacy in late-stage cancers. Taxane-based therapies in the context of other hormonal therapies or alone seem to have limited efficacy in breast and prostate cancers. However, in the case of ovarian cancer, where EMT and the gain of stem features by the cancer cells is common-place, large scale de-bulking of the tumor burden by surgical resection seems to have the greatest impact. Taxanes, platinum, and the like occupy the standard of care for late-stage cancer patients, but notoriously have short remission times. Thus, combining such traditional chemotherapy with targeted drugs or more recently immune checkpoint inhibition therapy seems to address the fast growing tumor cells, more quiescent, and those undergoing EMT.

Management of the anticancer drugs for patients based on neurotoxicity, the effects on canonical MAPK and EGFR signaling have been the focus of several studies. Phase II clinical trial analysis indicated that although toleration of CI-1040 was considerable, it failed to demonstrate sufficient antitumor activity [122]. The second generation MEK PD 0325901 showed significantly higher response in various tumors tested, including: breast, colon, pancreatic, and non-small-cell lung cancer. These findings were also related to breast cancer cell resistance to gefitinib, a EGFR tyrosine kinase inhibitor. Gefitinib successfully inhibited EGFR activation in SK-Br-3, MDA-MB-261, and MDA-MB-468 breast cancer cells, although p42/p44-MAPK and AKT phosphorylation was not reduced in MDA-MB-468 compared to the other cells [123]. PI3K inhibitor LY294002 or MEK inhibitor PD 98059 were more effective at reducing cells growth. Combination therapy resulted in apoptosis that was higher compared to single treatments. Overexpression of activated MAPK in normal mammary MCF-10A cells increased resistance, once again indicating the role of this signaling pathway for tumor progression. Epigenetics and transcriptional regulation also play a role in therapeutic efficacy. Promoter hypermethylation and silencing of a Wnt antagonist, secreted frizzled related protein 5 (SFRP5), was associated with ovarian cancer cell chemoresistance to cisplatin [124]. SFRP5 methylation was directly related to poor prognosis in patients who were treated with platinum-based chemotherapy. Cancer-promoting genes were activated based on the epigenetic silencing of Wnt-associated gene SFRP5. These findings link to the Twist- and AKT2-mediated events of EMT where cancer cell invasion, colony formation, and tumor growth were increased. Plant-based natural products such as Genistein, targets Akt/glycogen synthase kinase-3 (GSK-3) signaling [125]. The transgenic adenocarcinoma mouse prostate model (TRAMP/FVB) treated with Genistein had reduce prostate size and poorly defined tissue. Prostatic intra-epithelial neoplasia (PIN) was reduced using Genistein, and there was decreased Snail and migratory potential. Genistein is effective for treatment of various tumors and alters apoptosis, cell cycle and angiogenesis – further inhibiting metastasis. Specifically, caspases, Bcl-2, MAPK, Wnt, and AKT signaling are targeted with Genistein, while possessing synergy with other chemotherapy (*i.e.*, adriamycin, docetaxel, tamoxifen). Quercetin (EGCG) is reported to inhibit EMT, migration, and stem features [126]. The CD44+/CD133+ expressed prostate cancer cells decreased following EGCG treatment, indicating its ability to inhibit self-renewal. Caspase-3/7 activation was coupled with inhibition of Bcl-2, survivin, and Snail. Molecules responsible for invasion and migration were also inhibited and this agent may be useful in future therapy.

Natural products often have more than one target, which comes with advantages and disadvantages. However, the lack of rigorous studies on effective doses of such natural products has been the greatest obstacle to their wide adoption. The heterogeneous nature of the tumor and its microenvironment defines therapeutic efficacy. Mechanisms of inducing synthetic lethality targeting both compartments consider the plasticity of the disease. Ultimately, combination therapies that balance the blocking of resistance mechanisms of first-line therapies without compromising systemic functions are the obvious goal.

## 6. Conclusions

Studies discussed here indicate important mechanisms of cancer progression that are intimately linked to EMT progression in multiple solid tumors. Although the linking of EMT to the process of metastasis is not clear, its role in therapeutic resistance is evident. This is the result of the stem-like properties acquired by cells undergoing EMT as well as it being a means of potentiating tumor heterogeneity. The study of EMT benefits the strategies of cancer prevention, therapy, and prognosis.

## Abbreviations

EMT: Epithelial mesenchymal transition
TGF-β: Transforming growth factor β
EGF: Epidermal growth factor
MET: Mesenchymal-epithelial transition
MAPK: Mitogen activated protein kinase
ECM: Extracellular matrix
VEGF-A: Vascular endothelial growth factor-A
NED: Neuroendocrine differentiation
RANKL: Receptor activator of NFκB ligand
TME: Tumor microenvironment
PDGF: Platelet-derived growth factor
CAF: Cancer-associated fibroblast
NAF: Normal-associated fibroblast
FSP-1: Fibroblast-specific protein-1
LNCaP: Prostate cancer cells from left supraclavicular lymph node
PI3K: Phosphatidylinositol-3 kinase
IL-6: Pro-inflammatory cytokine interleukin-6
STC-1: Stanniocalcin-1
AR: Androgen receptor
ZEB-1: Zinc finger E-box-binding homeobox 1
ARCaP_M_: Androgen Repressed Metastatic Human Prostate Cancer Cell Line
UO126: MEK1 and MEK2 selective inhibitor
MMP: Matrix metalloproteinase
BMP: Bone morphogenetic protein
HGF: Hepatocyte growth factor
FGF: Fibroblast growth factor
CTC: Circulating tumor cells
PAR1: Protease-activated receptor-1
α-SMA: α-smooth muscle actin
MSC: Mesenchymal stem cells
ROS: Reactive oxygen species
HNSCC: Head and neck squamous cell cancer
OSCC: Oral squamous cell cancer
FAK: Focal adhesion kinase
CSC: Cancer stem cells
ALDH1: Aldehyde dehydrogenase 1
CXCR4: chemokine (C-X-C motif) receptor 4
uPA: Urokinase plasminogen activator
IGF: Insulin growth factor
OC: Osteocalcin
BSP: Bone sialoprotein
OPN: Osteopontin
OPG: Osteoprotegrin
PTEN: Phosphatase and tensin homolog
NSCLC: Non-small-cell lung cancer
